# Prescription Patterns of Wu Lin San Concentrated Extract Product for Cystitis in Taiwan: A Population-Based Study

**DOI:** 10.1155/2020/2605462

**Published:** 2020-05-20

**Authors:** Chia-Jung Lee, Steven Kuan-Hua Huan, Yi-Hui Lee, Yu-Shao Yeh, I-Hsin Lin, Ching-Chiung Wang

**Affiliations:** ^1^PhD Program in Clinical Drug Development of Herbal Medicine, College of Pharmacy, Taipei Medical University, Taipei, Taiwan; ^2^Graduate Institute of Pharmacognosy, College of Pharmacy, Taipei Medical University, Taipei, Taiwan; ^3^Traditional Herbal Medicine Research Center, Taipei Medical University Hospital, Taipei, Taiwan; ^4^Division of Urology, Department of Surgery, Chi Mei Medical Center, Tainan, Taiwan; ^5^Department of Biotechnology, Chia Nan University of Pharmacy Science, Tainan, Taiwan; ^6^Department of Traditional Chinese Medicine, Wan Fang Hospital, Taipei, Taiwan; ^7^School of Post-Baccalaureate Chinese Medicine, Tzu Chi University, Hualien, Taiwan; ^8^School of Pharmacy, College of Pharmacy, Taipei Medical University, Taipei, Taiwan

## Abstract

The indications for the concentrated extract product (CEP) of Wu Lin San (WLS) are urethritis, cystitis, and gonorrhea. In clinical settings, WLS is combined with other CEPs used. However, there are no prescribed guidelines of CEPs in Taiwan. In this study, we would establish the CEP-prescribed applications of WLS for cystitis according to the clinical prescription patterns and ancient traditional medicine books. The prescription patterns of WLS were analyzed from the National Health Insurance Research Database of Taiwan for the period from 2000 to 2015. The results show that WLS was most frequently prescribed for cystitis (17.12% of a total prescriptions), and its prescribed dosage was 3∼5 g per day. Among them, 62.53% were for patients >40 years, and 72.45% were for women. Moreover, prescription patterns of WLS for cystitis were divided into 4 types: Type 1, WLS combined with Pa Cheng San (PCS) and Ti Tang Tang (29.75%); Type 2, WLS combined with PCS and dandelion (13.89%); Type 3, WLS combined with PCS and Tao Ho Cheng Chi Tang (6.63%); and Type 4, WLS combined with PCS (2.75%). According to lectures, review revealed the following principles of WLS application. WLS only should be adopted for simple heat strangury, while Type 4 should be applied for excess heat and dampness strangury. For patients with heat strangury coupled with an early-stage blood amassment pattern in *lower jiao* (abdomen), Type 3 could be administered. Type 2 should be used for heat strangury accompanied by dampness toxicity with infection. By contrast, Type 1 should be applied to patients with severe blood stasis. The application principles of WLS with other CEPs could serve as a reference for cystitis treatment in clinical settings.

## 1. Introduction

In the practice of traditional Chinese medicine (TCM), physicians prescribe medication to patients according to their conditions and prescription patterns involving various medicinal ingredients. Patients then boil the medicinal ingredients together to obtain a decoction, which is consumed orally. Currently, TCM physicians in Taiwan mostly prescribe the concentrated extract products (CEP) of Chinese medicine in granule form [1]. Patients could consume the mixed powder orally without decocting it. Although physicians still prescribe TCM according to traditional prescription patterns, the composition of current prescriptions has become different from that of traditional ones, which consist of individual medicinal ingredients [2]. This study analyzed the prescription patterns of the CEP of Wu Lin San (WLS) followed by TCM physicians in clinical settings by using data from the National Health Insurance Research Database (NHIRD) of Taiwan.

WLS was recorded in *Tai Ping Huei Min Ho Chi Chu Fang* during the Song dynasty. It is composed of Zhi Zi (*Gardenia jasminoides*), Fu Ling (*Poria cocos*), Dang Gui (*Angelica sinensis*), Chi Shao (*Paeonia lactiflora*), Deng Xin Cao (*Juncus effusus*), and Gan Cao (*Glycyrrhiza uralensis*) [3]. These medicinal ingredients mainly perform heat clearance, diuresis, and blood cooling functions (Table 1) [4]. According to the theory of TCM prescription patterns, medicinal ingredients clustered in a prescription can be assigned any of the following four roles based on their functions, sovereign, minister, assistant, and courier [5]. For WLS, Zhi Zi serves as the sovereign ingredient because it performs the main functions of heat clearance and diuresis. Fu Ling and Chi Shao are the minister ingredients: Fu Ling facilitates dampness elimination and enhances the diuresis effect of Zhi Zi; Chi Shao performs heat clearance and blood cooling functions, thereby improving the heat dissipation and analgesic effects of Zhi Zi. Dang Gui and Deng Xin Cao are the assistant ingredients: Dang Gui can increase the blood supply and accelerate blood circulation, alleviating pain, and can regulate the medicinal properties of Zhi Zi, Fu Ling, and Chi Shao; Deng Xin Cao can clear heart fire and facilitate dampness elimination, decreasing heat. Finally, Gan Cao serves as the courier ingredient because it facilitates the integration of all other ingredients into a prescription to be used for treating strangury in an interior heat deficiency pattern [4]. However, a traditional medical doctor follows eight principles (*ba gang*), the general guideline of TCM, for diagnostic differentiation of syndromes. The eight principles refer to Yin(陰), Yang(陽), Exterior(表), Interior(裏), Cold(寒), Heat(熱), Deficiency(虛), and Excess(實). According to the eight principles, herbs in WLS could be classified as follows: Zhi Zi, Chi Shao, and Deng Xin Cao as interior-heat-excess syndrome and Fu Ling, Dang Gui, and Gan Cao as deficiency syndrome. Taken together, WLS is a formula to use in interior-heat-excess (deficiency) syndrome.

This formula is used for treating internal heat in the bladder, dysuria, acute pain in the umbilicus and abdomen, fatigue, urinary gravel, dark colored urine, and hematuria [6]. Therefore, the indications for the CEP of WLS are urethritis, cystitis, vesical calculi, renal calculi, and gonorrhea [3]. In addition to prescribing WLS alone for treating urinary-tract-related diseases, Taiwanese TCM physicians prescribe WLS along with other prescriptions in clinical practice. Whether general rules exist with regard to the addition of multiple prescriptions to WLS and whether such prescription patterns remain compatible with the basic theoretical principles of TCM warrant further investigation.

The CEPs of Chinese medicine have been adopted in Taiwan since 1952, and their application has been covered by the NHI program since 1996. The NHIRD contains the TCM usage records of almost the entire Taiwanese population (≥99%); thus, this database has excellent sample representativeness. Using the data collected from the NHIRD, numerous recent studies have analyzed TCM prescription patterns commonly applied to treat specific diseases [7–9]. To investigate the prescription patterns of WLS, the present study collected outpatient data related to TCM usage from the NHIRD. First, the disease for which WLS was the most commonly prescribed was determined using International Classification of Diseases, Ninth Revision, Clinical Modification (ICD-9-CM) codes. Subsequently, within the scope of this disease, the prescription patterns used, in which WLS was combined with other prescriptions, were discussed to identify the differences in their clinical applications to various symptom patterns.

## 2. Materials and Methods

### 2.1. Eight Principle Classification of Herbs in WLS

Classification of herbs in WLS, including Zhi Zi (*G*. *jasminoides*), Fu Ling (*P*. *cocos*), Dang Gui (*A*. *sinensis*), Chi Shao (*P*. *lactiflora*), Deng Xin Cao (*J*. *effusus*), and Gan Cao (*G*. *uralensis*), into eight principles was according to the Professor Kun-Ying Yen's Book [10].

### 2.2. Prescriptions Patterns of WLS in NHIRD

This study was conducted using the National Health Insurance Research Database of Taiwan, which contains the data of 1,000,000 beneficiaries sampled for the period from 2000 to 2015. This database contains all inpatient and outpatient records, pharmacy service records, and medication prescription records. We collected the WLS prescription records for the period of 2000 to 2015, and the diagnosis codes were also acquired. The frequency of the diagnosis codes was estimated based on the first three digits of the ICD-9-CM code.

WLS prescriptions were subdivided into three groups based on the daily dosage: <3 g, 3∼5 g, and >5 g per day. The most prevalent diagnosis was chosen; subsequently, the prescription pattern of that diagnosis was analyzed, focusing on the combination of WLS with other drugs in any given prescription.

## 3. Results

### 3.1. Disease Frequency Distribution for WLS Prescriptions

The outpatient data related to TCM usage from the NHIRD were analyzed. The results revealed the top 10 diseases for which WLS was prescribed at the three daily dosages (Table 2). Urinary-tract-related diseases such as cystitis, urethritis, and prostatitis (ICD-9-CM codes 592, 595, 597, 599, 601, and 788) comprised most of the diseases, followed by vaginal discomfort and genital tract bleeding in women (ICD-9-CM codes 623 and 626). Cystitis (ICD-9-CM code 595) ranked first in the list of diseases for which WLS was prescribed at a daily dosage of more than 3 g. Moreover, WLS was the most prescribed for cystitis (ICD-9-CM code 595). Approximately 17.72% of a total of 104,407 WLS prescriptions were for cystitis, and the most prescribed dosage was 3∼5 g per day (12,041/17,875, 67.38%) (Table 2).

### 3.2. Age-Specific Frequency Distribution of Cystitis Treated with WLS

Based on the aforementioned data, the prescription pattern of WLS for cystitis (ICD-9-CM code 595) was assessed according to patients' age. As shown in Table 3, most patients were women aged 40 years and older. Of 17,875 WLS prescriptions for cystitis, 62.53% were for patients aged more than 40 years, and 72.45% were for women. Age and gender are indeed important factors for cystitis.

### 3.3. Prescription Pattern of WLS for Cystitis

The prescription patterns of WLS for cystitis were divided into four types according to daily dosage 3∼5 g group (Table 4): Type 1, WLS combined with Pa Cheng San (PCS, 八正散) and Ti Tang Tang (TTT, 抵當湯) (3,582/12,041, 29.75%); Type 2, WLS combined with PCS and dandelion (*Taraxacum mongolicum*) (1,673/12,041, 13.89%); Type 3, WLS combined with PCS and Tao Ho Cheng Chi Tang (THCCT, 桃核承氣湯) (798/12,041, 6.63%); Type 4, WLS combined with PCS (331/12,041, 2.75%). However, WLS alone was not the top five but still had a very high percentage to treat cystitis. The results proved that the major indication for WLS is cystitis and that WLS is usually combined with PCS (more than 54.4%). The results presented important information that when WLS was treated for cystitis, the prescription used some specific rules in Taiwan.

TTT, composed of Da Huang, Tao Ren, Shui Chih, and Meng Chung, typically used to treat blood clots, anxiety, irritability, and heat during menstruation. Because that the elderly women were the most vulnerable to cystitis, TTT is often prescribed with WLS and PCS. In this study, the application rate of this prescription pattern was the highest at 29.75% (Type 1), followed by WLS in combination with dandelion (Type 2) and with THCCT (Type 3). The application of these prescription patterns was inferred to be related to the chief complaints of patients. Cystitis is often accompanied by pain. Depending on the severity of pain, Type 1, Type 2, and Type 3 were applied to treat severe, moderate, and mild symptoms, respectively. However, if the chief complaint was a burning sensation, Type 2 would be selected first. Based on the study results, we inferred that TCM physicians interpreted that most cases of cystitis (ICD-9-CM code 595) were cystitis-coupled with other symptoms, compound prescriptions derived from prescription patterns were adopted for treating these cases (Table 1).

Cystitis is the medical term for inflammation of the bladder. Most of the time, the inflammation is caused by a bacterial infection. A bladder infection can be painful and annoying, and it can become a serious health problem if the infection spreads to kidneys and other urinary-tract-related disease. All these 4 types of prescription patterns for cystitis treated with WLS are shown in Table 5. Cystitis, more close to TCM theory, was when patients with heat strangury, caused by heat evil causing low quantities of urine, a high frequency of urination, and pain during urinary discharge, and even with solid, heat, and damp strangury symptoms. Further, following the eight principles, we classified each medicinal ingredient of these four types of prescription patterns. For exterior and interior principles, most of these drugs presented similar characteristic as being interior, but Gan Cao and Kuei Chih did not show characteristic of being exterior nor interior. For cold and heat principles, only Dang Gui and Kuei Chih presented to treat cold property. Others were treated with hot property. For deficiency and excess principles, Fu Ling, Dang Gui, Gan Cao, and Kuei Chih could use as deficiency syndrome. Taken together, most of these drugs presented similar characteristic with interior-heat-excess syndrome. However, Fu Ling, Dang Gui, Gan Cao, and Kuei Chih presents deficiency syndrome.

### 3.4. Frequency Distribution of TCMs for Cystitis

The frequencies of TCM prescriptions for cystitis were 25.61% for WLS, 14.75% for PCS, 6.11% for TTT, 2.06% for Lung Tan Hsieh Kan Tang (LTHKT, 龍膽瀉肝湯), and 1.17% for Chu Ling Tang (CLT, 豬苓湯) among a total of 69,808 prescriptions. Among the months, summer (June to August) was the season in which cystitis often occurs (Table 6).

## 4. Discussion

WLS and PCS were both recorded in *Tai Ping Huei Min Ho Chi Chu Fang* as applicable for treating heat strangury. The main difference between them is that WLS can be used to treat kidney qi deficiency, because of the drugs Fu Ling, Dang Gui, and Gan Cao, whereas PCS is applied to treat interior excess heat and dryness in the mouth and throat. In comparison, TTT and THCCT were both recorded in *Shang Han Lun* as applicable for treating blood amassment in *lower jiao* (i.e., abdomen). THCCT, because of Da Huang and Mang Hsiao, is used to treat severe constipation, whereas TTT, because of Shui Chih and Meng Chung, is used to treat severe blood stasis.

**Figure 1:**
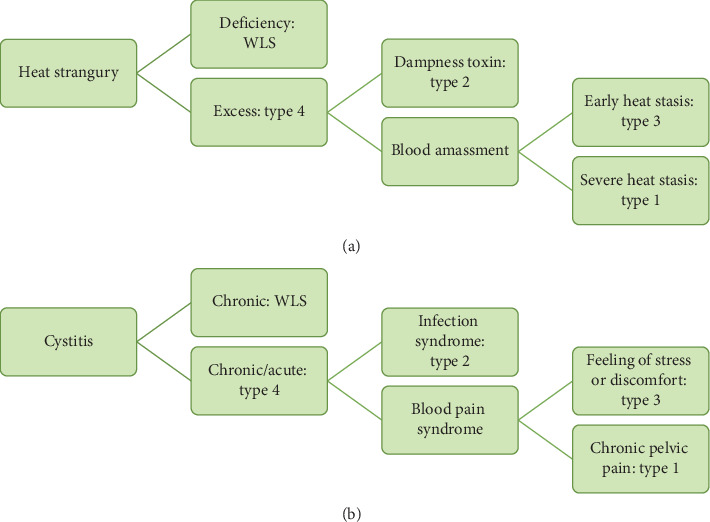
Principles of WLS application. Principles according to the traditional Chinese medicine diagnoses (a) and Western medicine diagnoses (b).

In summary of the traditional applications of WLS and the derived common prescription patterns currently applied by Taiwanese TCM physicians, cystitis symptoms can be interpreted and treated in accordance with the following principles. WLS should be adopted to treat patients who report heat strangury caused by heat evil and who exhibit low quantities of urine, a high frequency of urination, and pain during urinary discharge, which are similar to the cystitis symptoms described in Western medicine, and those with simple heat strangury and lower back soreness. For patients with solid, heat, and damp strangury whose urine color is relatively dark (turbid or with blood), Type 4 should be used. Further, Type 3 should be used to treat patients with solid, heat, and damp strangury coupled with early-stage blood amassment, pain, and swelling in *lower jiao* and severe constipation. However, for the symptom with conspicuous infection constituting a damp toxicity pattern, Type 2 could be used to treat patients of cystitis with solid, heat, and damp strangury coupled. Finally, Type 1 should be used to treat patients with severe blood amassment (Figure 1(a)).

Clinical trials of Chinese medicine have indicated that WLS, in which a few medicinal ingredients may be added to or removed from the original prescription, is effective for alleviating acute urinary tract infections [11–13] and urethral calculi [14]. PCS has also been verified as an effective medicinal ingredient for treating chronic prostatitis [15]. Furthermore, WLS and PCS were combined to prepare water pan pills (Shui Fan Wan), which were then administered to 138 patients with urinary tract infections. The inclusion criteria were damp heat strangury presenting as lower back pain, high-frequency urinary discharge, urinary urgency, or difficulty in urinary discharge. The results showed that 77.5% of the patients experienced symptom mitigation [16]. That study supported the combination of WLS with PCS (Type 4). Another study investigated the treatment effect of PCS combined with THCCT on type III chronic prostatitis. Sixty patients with pelvic pain or discomfort, high-frequency urinary discharge, urinary urgency, urinary pain, difficulty in urinary discharge, or testicular falling or bulging pain were recruited as participants. The participants were randomly grouped and treated; 80.6% of them experienced improvement in their symptoms, evidencing the effectiveness of Type 3 usage [17]. Two case studies have determined that although PCS should be adopted to treat patients with acute cystitis because the disease manifests as water collection, THCCT, which should be applied to blood collection, can also cure patients with this disease. Wei and Wang inferred that THCCT facilitates bowel emptying, and heat stasis can be dissipated through this process, thereby reducing abdominal pressure and improving blood circulation in the organs [18]. Therefore, THCCT can be combined with PCS to treat acute cystitis accompanied by blood collection. With Type 2, in a clinical test exploring the effect of PCS combined with a substantial dandelion amount on damp heat-type urinary tract infection, 90% of the 60 recruited patients showed improvement; specifically, the white blood cell count and the number of bacterium colonies were reduced in the urine [19]. That study supported that the combination of dandelion with PCS strengthens bacteria suppression and facilitates heat clearance and detoxification [20]. For Type 1 and Type 3, THCCT and TTT are both recorded in *Shang Han Lun* as applicable for treating blood amassment in the Tai Yang meridian. The difference between them is the disease phase during which they should be applied. Early-stage blood collection in the Tai Yang meridian is characterized by heat in the bladder or *lower jiao*, whereas the later-phase condition is characterized by interior heat stasis. Early- and later-stage blood amassment should be treated with THCCT and TTT, respectively [21]. In addition, TTT has been proven to be effective for ameliorating difficulty in urinating caused by prostate enlargement [22], supporting the application of TTT to cystitis.

With reference to the aforementioned clinical studies, Types 1–4 prescription patterns of WLS can be explained under the framework of the sovereign, minister, assistant, and courier theory. WLS can clear away heat and promote diuresis; thus, it serves as the sovereign formula in a prescription. Similar to Fu Ling, PCS enhances the diuresis effect of WLS and eliminates *lower jiao* dampness and thus serves as the minister ingredient. Similar to Chi Shao, TTT and THCCT can eliminate blood stasis by promoting blood circulation and thus serve as minister ingredients. Moreover, TTT and THCCT exert effects similar to those of Deng Xin Cao and thus can serve as assistant ingredients. Dandelion exerts effects similar to those of Deng Xin Cao; hence, it is categorized as an assistant ingredient (Table 1).

Few studies have investigated TCM prescriptions. Dandelion extracts have been verified to be effective for suppressing Lipopolysaccharide-induced inflammatory responses in RAW264.7 cells and for suppressing inducible nitric oxide synthase and cyclooxygenase-2 expression [23, 24]. Zhi Zi and Deng Xin Cao are used in WLS and PCS. Both ingredients have been shown to suppress LPS-induced inflammatory responses in RAW264.7 cells [25–28]. Zhi Zi contains glycoprotein, which has antioxidant and anti-inflammatory effects; it also contains iridoid glycosides and crocetin glycosides, which can suppress NO formation [27]. Iridoid glycosides can suppress nuclear factor (NF)-*κ*B and can activate mitogen-activated protein kinase (MAPK) signaling pathways by regulating Toll-like receptor 4 expression [28]. Moreover, Zhi Zi extract can suppress histamine release [29], thereby achieving an antiallergy effect. Deng Xin Cao extract has been proven to have an anti-inflammatory effect [26], and phenanthrenoids have been identified as the main effective ingredient that suppresses NO formation [30, 31]. Tao Ren (*Prunus persica*) and Da Huang (*Rheum palmatum*) are two common medicinal ingredients in TTT and THCCT. The methanol extract of Tao Ren has been found to contain cyanogenic glycosides and phenolic compounds. Phenolic compounds can achieve antiallergy and anti-inflammatory effects by suppressing histamine release and the proinflammatory cytokines of tumor necrosis factor-alpha and interleukin-6 in human mast cells [32]. By contrast, cyanogenic glycosides can inhibit the Epstein-Barr virus early antigen activation induced by tumor promoter [33]. A main ingredient group of Da Huang, anthraquinones, has anti-inflammatory effects [34]. Their key ingredients, emodin [35], and aloe emodin [36], achieve anti-inflammatory effects by suppressing NF-*κ*B, MAPK, and PI3K pathways. Another study indicated that stilbenes in Da Huang can inhibit platelet aggregation [37]. In summary, these commonly used medicinal ingredients have anti-inflammatory, antiallergy, and platelet-aggregation-inhibiting effects and are therefore effective for alleviating cystitis symptoms.

The theory of TCM indicates that cystitis is a manifestation of dampness and heat perfusion in the *lower jiao*. Hence, heat clearance medicine and diuretics are commonly applied as clinical treatments. Table 4 lists the TCM prescriptions commonly used to treat cystitis and the three dosages commonly prescribed. At a low dosage (<3 g per day), Che Qian Zi (*Plantago asiatica*), dandelion, Bai Mao Gen (*Imperata cylindrica*), Huang Bo (*Phellodendron amurense*), and Gan Cao (*Glycyrrhiza uralensis*) were ranked as the top five; at a medium dosage (3∼5 g per day), PCS, WLS, CLT, Dao Chi San (DCS, 導赤散), and LTHKT were ranked as the top five; and at a high dosage (>5 g per day), PCS, LTHKT, WLS, CLT, and DCS were ranked as the top five. Based on the study results, we inferred that PCS and LTHKT are more likely to be used alone as the primary daily prescription for cystitis because they have relatively strong heat clearance and diuresis effects. By contrast, WLS and TTT have relatively weak heat clearance and diuresis effects and are thus less likely to be used alone in prescriptions. They often must be used in combination with other compounds or single prescriptions. For example, to enhance the heat clearance effect, a combination of dandelion, Bai Mao Gen, and Huang Bo is often used; to strengthen the diuresis effect, a combination of Che Qian Zi and PCS is often used (Table 6).

## 5. Conclusions

**Figure 2:**
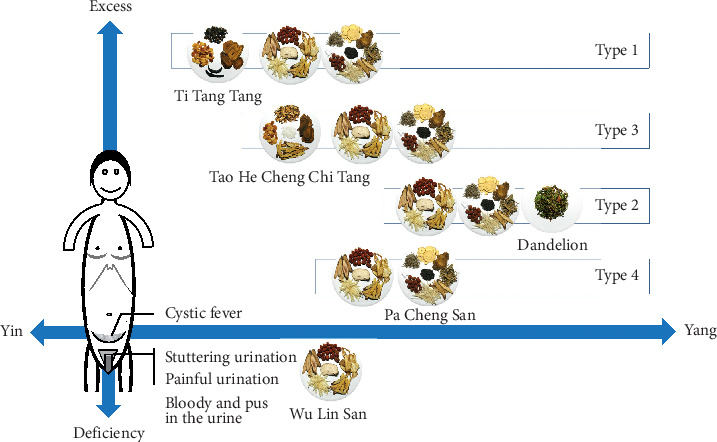
According to the Yin-Yang theory, the prescription patterns of WLS with other CEPs for cystitis.

The four common prescription patterns of WLS should be applied based on the interpretation of the condition according to TCM theory. The application principles are listed in Figure 1(a). The effects of individual prescription patterns are summarized in Figure 1(b). In this figure, the corresponding disease names in Western medicine have also been provided to identify the applicable diseases. Chronic cystitis can be treated with Type 5; chronic or acute cystitis can be treated with Type 4; interstitial cystitis along with conspicuous bladder pain and a sense of oppression or discomfort can be treated with Type 3; cystitis along with bacterial infections or notable inflammation can be treated with Type 2; and chronic pelvic pain can be treated with Type 1. Cystitis according to the Yin-Yang disease pattern could be treated with the compatibility of WLS with other CEPs as Figure 2. The above suggested principles could serve as a reference for the application of WLS prescription patterns to treat cystitis, in which WLS is combined with other CEPs. Moreover, the prescription patterns can be used for treating diseases interpreted according to Western medicine knowledge in reference to TCM theory.

## Figures and Tables

**Table 1:** Components of WLS and their roles in prescription patterns of WLS.

WLS				Role of ingredient	Prescription pattern			
Name	Scientific name	Used part	Dosage (g)		Type 1	Type 2	Type 3	Type 4
Zhi Zi	*Gardenia jasminoides*	Sclerotium	4	Sovereign	WLS^a^	WLS	WLS	WLS
Fu Ling	*Poria cocos*	Root	6	Minister	PCS^b^	PCS	PCS	PCS
Chi Shao	*Paeonia lactiflora*	Root	4	Minister	TTT^c^	–	THCCT^d^	–
Dang Gui	*Angelica sinensis*	Root	4.8	Assistant	–	–	–	–
Deng Xin Cao	*Juncus effuses*	Fruit	2	Assistant	TTT	Dandelion	THCCT	–
Gan Cao	*Glycyrrhiza uralensis*	Stem	4.8	Courier	–	–	–	–

^a^WLS: Wu Lin San. ^b^PCS: Pa Cheng San. ^c^TTT: Ti Tang Tang. ^d^THCCT: Tao Ho Cheng Chi Tang.

**Table 2:** Top 10 diseases for which WLS^a^ is prescribed at three daily dosages (according to the frequency distribution of ICD-9-CM codes).

No.	Code	All		Code	Dosage <3 g per day^b^		Code	Dosage 3∼5 g per day^c^		Code	Dosage >5 g per day^d^	
		Disease	%		Disease	%		Disease	%		Disease	%
1	595	Cystitis	17.72	597	Urethritis, not sexually transmitted, and urethral syndrome	10.57	595	Cystitis	30.64	595	Cystitis	18.21

2	597	Urethritis, not sexually transmitted, and urethral syndrome	11.06	788	Symptoms involving urinary system	8.67	597	Urethritis, not sexually transmitted, and urethral syndrome	8.72	597	Urethritis, not sexually transmitted, and urethral syndrome	16.17

3	788	Symptoms involving urinary system	9.40	595	Cystitis	8.52	788	Symptoms involving urinary system	7.24	788	Symptoms involving urinary system	12.05

4	780	General symptoms	3.85	780	General symptoms	7.78	780	General symptoms	3.78	599	Other disorders of urethra and urinary tract	3.26

5	626	Disorders of menstruation and other abnormal bleeding from female genital tract	3.65	786	Symptoms involving respiratory system and other chest symptoms	3.78	626	Disorders of menstruation and other abnormal bleeding from female genital tract	2.69	581	Nephrotic syndrome	3.22

6	724	Other and unspecified disorders of back	2.75	784	Symptoms involving head and neck	3.40	536	Disorders of function of stomach	2.59	592	Calculus of kidney and ureter	3.14

7	623	Noninflammatory disorders of vagina	2.38	536	Disorders of function of stomach	3.04	623	Noninflammatory disorders of vagina	2.22	623	Noninflammatory disorders of vagina	3.09

8	599	Other disorders of urethra and urinary tract	2.29	477	Allergic rhinitis	2.37	724	Other and unspecified disorders of back	2.20	780	General symptoms	2.95

9	536	Disorders of function of stomach	2.10	724	Other and unspecified disorders of back	2.13	564	Functional digestive disorders, not elsewhere classified	2.08	601	Inflammatory diseases of prostate	2.70

10	592	Calculus of kidney and ureter	1.92	626	Disorders of menstruation and other abnormal bleeding from female genital tract	1.99	599	Other disorders of urethra and urinary tract	1.48	724	Other and unspecified disorders of back	2.25

^a^WLS : Wu Lin San. ^b^Number of prescriptions for dosage <3 g per day (not including 3 g): *n* = 1,330. ^c^Number of prescriptions for dosage 3∼5 g per day: *n* = 12,041. ^d^Number of prescriptions for dosage >5 g per day (not including 5 g): *n* = 4,504.

**Table 3:** Age-specific frequency distribution of cystitis treated with WLS^a^.

Age (years)	Prescription number/frequency (%)					
	Female		Male		Total	
0∼20	2,592	3.71	2,481	3.55	5,073	7.26
21∼30	6,505	9.32	1,927	2.76	8,432	12.08
31∼40	9,370	13.42	3,282	4.70	12,652	18.12
41∼50	12,142	17.39	3,747	5.37	15,889	22.76
>50	19,975	28.61	7,787	11.16	27,762	39.77
Total	50,584	72.45	19,224	27.54	69,808	100.00

^a^WLS: Wu Lin San.

**Table 4:** Four types of prescription patterns for cystitis treated with WLS.

Chinese herbal medicine	Daily dosage <3 g	Daily dosage 3∼5 g	Daily dosage >5 g
WLS^a^ + PCS^b^ + TTT^c^	118	3,582	≦5
WLS + PCS + Dandelion	89	1,673	210
WLS + PCS + THCCT^d^	56	798	0
WLS + PCS	30	331	271
WLS + Hua Shi Cao + Huang qin	≦5	94	426
WLS	≦5	20	381
WLS + Hua Shi Cao	0	≦5	250
WLS + PCS + DCS^e^	≦5	43	96
WLS + PCS + GZFLW^f^	≦5	124	0
WLS + LTHKT^g^	13	40	63
Total	1,324	12,041	4,504

^a^WLS: Wu Lin San. ^b^PCS: Pa Cheng San. ^c^TTT: Ti Tang Tang. ^d^THCCT: Tao Ho Cheng Chi Tang. ^e^DCS: Dao Chi San. ^f^GZFLW: Gui Zhi Fu Ling Wan. ^g^LTHKT: Lung Tan Hsieh Kan Tang.

**Table 5:** Four types of prescription patterns^a^ for cystitis treated with WLS.

Formula	WLS^b^	Type 4		Type 3			Type 2			Type 1		
		WLS	PCS^c^	WLS	PCS	THCCT^d^	WLS	PCS	Dandelion	WLS	PCS	TTT^e^
Gan Cao	D	D		D		D	D			D		
Fu Ling	I-D	I-D		I-D			I-D			I-D		
Dang Gui	I-C-D	I-C-D		I-C-D			I-C-D			I-C-D		
Chi Shao	I-H-Ex	I-H-Ex		I-H-Ex			I-H-Ex			I-H-Ex		
Deng Xin Cao	I-H-Ex	I-H-Ex	I-H-Ex	I-H-Ex	I-H-Ex		I-H-Ex	I-H-Ex		I-H-Ex	I-H-Ex	
Zhi Zi	I-H-Ex	I-H-Ex	I-H-Ex	I-H-Ex	I-H-Ex		I-H-Ex	I-H-Ex		I-H-Ex	I-H-Ex	
Mu Tung			I-H-Ex		I-H-Ex			I-H-Ex			I-H-Ex	
Da Huang			I-H-Ex		I-H-Ex	I-H-Ex		I-H-Ex			I-H-Ex	I-H-Ex
Chu Mai			I-H-Ex		I-H-Ex			I-H-Ex			I-H-Ex	
Pien Fan			I-H-Ex		I-H-Ex			I-H-Ex			I-H-Ex	
Hua Shih			I-H-Ex		I-H-Ex			I-H-Ex			I-H-Ex	
Gan Cao Shao			D		D			D			D	
Che Qian Zi			I-H-Ex		I-H-Ex			I-H-Ex			I-H-Ex	
Tao Ren						I-Ex						I-Ex
Kuei Chih						C						
Mang Hsiao						I-H-Ex						
Dandelion									I-H-Ex			
Shui Chih												I-Ex
Meng Chung												I-H-Ex

^a^Total number of prescriptions, *n* = 17,875. ^b^WLS: Wu Lin San. ^c^PCS: Pa Cheng San. ^d^THCCT: Tao Ho Cheng Chi Tang. ^e^TTT: Ti Tang Tang. Eight principles: Exterior (E), Interior (I), Cold (C), Heat (H), Deficiency (D) and Excess (Ex).

**Table 6:** Top 10 Chinese herbal medicines prescribed at different month for cystitis treatment.

Drug name	Month												
	July	October	June	August	December	November	September	May	March	April	January	February	Total
Wu Lin San	1,767	1,744	1,632	1,567	1,662	1,555	1,530	1,419	1,321	1,267	1,292	1,119	17,875
	2.53%	2.50%	2.34%	2.24%	2.38%	2.23%	2.19%	2.03%	1.89%	1.81%	1.85%	1.6%	25.61%
Pa Cheng San	1,024	1,037	927	835	1,084	929	888	844	690	644	775	622	10,299
	1.47%	1.49%	1.33%	1.20%	1.55%	1.33%	1.27%	1.21%	0.99%	0.92%	1.11%	0.89%	14.75%
Ti Tang Tang	383	471	366	338	525	428	379	312	262	212	371	218	4,265
	0.55%	0.67%	0.52%	0.48%	0.75%	0.61%	0.54%	0.45%	0.38%	0.30%	0.53%	0.31%	6.11%
Dandelion	245	231	268	222	319	249	221	284	224	207	195	224	2,889
	0.35%	0.33%	0.38%	0.32%	0.46%	0.36%	0.32%	0.41%	0.32%	0.30%	0.28%	0.32%	4.14%
Lung Tan Hsieh Kan Tang	145	123	129	147	111	120	140	106	105	112	105	98	1,441
	0.21%	0.18%	0.18%	0.21%	0.16%	0.17%	0.20%	0.15%	0.15%	0.16%	0.15%	0.14%	2.06%
Dao Chi San	151	121	133	120	89	115	115	112	105	113	101	92	1,367
	0.22%	0.17%	0.19%	0.17%	0.13%	0.16%	0.16%	0.16%	0.15%	0.16%	0.14%	0.13%	1.96%
Chu Ling Tang	131	125	119	109	79	98	87	103	99	103	72	69	1,194
	0.19%	0.18%	0.17%	0.16%	0.11%	0.14%	0.12%	0.15%	0.14%	0.15%	0.10%	0.10%	1.71%
Che Qian Zi	125	99	100	109	62	94	106	84	88	98	62	63	1,090
	0.18%	0.14%	0.14%	0.16%	0.09%	0.13%	0.15%	0.12%	0.13%	0.14%	0.09%	0.09%	1.56%
Hua Shi Cao	75	114	84	89	116	90	65	78	118	75	66	66	1,036
	0.11%	0.16%	0.12%	0.13%	0.17%	0.13%	0.09%	0.11%	0.17%	0.11%	0.09%	0.09%	1.48%
Tao Ho Cheng Chi Tang	86	133	84	114	98	87	123	74	50	67	71	32	1,019
	0.12%	0.19%	0.12%	0.16%	0.14%	0.12%	0.18%	0.11%	0.07%	0.10%	0.10%	0.05%	1.46%

Total number of prescriptions: *n* = 69,808; Bottom line: single herb.

## Data Availability

All data analyzed during this study are included in this manuscript.
